# Oregano powder substitution and shelf life in pork chorizo using Mexican oregano essential oil

**DOI:** 10.1002/fsn3.668

**Published:** 2018-05-15

**Authors:** Yessica J. Perales‐Jasso, Stephannie A. Gamez‐Noyola, Juana Aranda‐Ruiz, Carlos A. Hernandez‐Martinez, Guadalupe Gutierrez‐Soto, Alejandro I. Luna‐Maldonado, Ramon Silva‐Vazquez, Michael E. Hume, Gerardo Mendez‐Zamora

**Affiliations:** ^1^ Facultad de Agronomia Universidad Autonoma de Nuevo Leon General Escobedo Mexico; ^2^ Instituto Tecnologico de Parral Hidalgo del Parral Mexico; ^3^ Food and Feed Safety Research Unit Southern Plains Agricultural Research Center U.S. Department of Agriculture Agricultural Research Service College Station Texas

**Keywords:** antioxidant, color, essential oil, Mexican oregano, microbiology, oregano powder, pH, sausage, sensory, sensory evaluation, textural profile

## Abstract

The aim of this study was to evaluate the effect of oregano essential oil (MOO) from Mexican oregano, *Lippia berlandieri* Schauer, as substitute for Mexican oregano powder (MOP) on pork chorizo physicochemical characteristics, texture, antioxidant capacity, aerobic bacteria colony counts, and sensory evaluation under storage conditions over 7 d. The treatments were T1 = chorizo + 0.1% MOP and T2 = chorizo + 0.1% MOO. The pH, redness (a*), yellowness (b*), Chroma, and browning index (BI) were affected by treatments and storage time. T2 presented lower pH (5.27) at d 1 than at d 7 (5.34), as well as a* (23.13 vs. 25.27), b* (14.85 vs. 17.45), Chroma (28.60 vs. 30.79), and BI (103.42 vs. 109.82) were higher at d 7. At d 1, hardness (1392.75 vs. 872.29 g), springiness (0.3675 vs. 0.3351 mm), gumminess (491.45 vs. 284.38 g), and chewiness (180.25 vs. 95.43 g mm) were higher in T1 than T2. Aerobic bacteria counts (T1—4.19 vs. 4.73 log CFU/g and T2—4.37 vs. 4.50 log CFU/g, respectively) increased within each treatment at d 7. Antioxidant capacity was not affected (26.48 and 27.42%). Oregano odor was different at 7 d with T2 having a stronger odor (5.70) than T1 with oregano powder (4.63). Mexican oregano essential oil in the pork chorizo formulation improved pH, color parameters, textural profile, and sensory characteristics.

## 1 INTRODUCTION

Pork meat is an important component in the human diet and is a major commodity in the meat industry for the manufacture of sausage, ham, and chorizo. Chorizo is a raw sausage composed of beef, pork meat and fat, additives, and spices that give it its characteristic flavor (Porcella et al., 2001). Chorizo preparation is still basically a family art employing techniques requiring rudimentary utensils and natural casings. The sausages are hand‐kneaded and stuffed without any aseptic measures being taken (González & Díez, 2002). In Mexico, chorizo is commonly manufactured from pork meat and is classified as a fresh sausage product and comes in a variety of formulations and manufacture. This Mexican product has a pork meat content of 70%–80% and back fat content of 15%–20%. The color of Mexican chorizo is characteristically red and is seasoned with spices, salt, oregano powder, and vinegar.

Consumers today want foods containing healthy ingredients that have good composition and an extended shelf life accompanied by minimal deterioration in components and sensory characteristics. Studies have been carried out with chorizo analyzing its physical characteristics (Gimeno, Ansorena, Astiasarán, & Bello, 2000), refrigeration stability (Porcella et al., 2001), microbial quality (Casquete et al., 2012; González & Díez, 2002), biochemical parameters (Salgado, García‐Fortán, Franco, López, & Carballo, 2006), polycyclic aromatic hydrocarbons content (Lorenzo et al., 2011), low molecular weight antioxidant components (Broncano, Otte, Petrón, Parra, & Timón, 2012), and textural, color, moisture, and morphological characteristics of chorizos encased in natural and synthetic casings (Ledesma, Laca, Rendueles, & Díaz, 2016). However, Mexican oregano essential oil (MOO) has not been used and evaluated in the chorizo pork formulation as a substitute for oregano powder. Mexican oregano essential oil may serve as an enhancement over Mexican oregano powder (MOP) as alternative in pork chorizo manufacture by improving conservation of nutrients as well as by reducing potential contaminants introduced with oregano powder or raw materials (González‐Tenorio et al., 2013; Hajmeer, Basheer, Hew, & Cliver, 2006; Hew, Hajmeer, Farver, Glover, & Cliver, 2005; Hew et al., 2006).

Mexican oregano, *Lippia berlandieri* Schauer, essential oil (MOO) is characterized by its strong odor and different biological activities, and the plant yields high quantities of essential oils (Avila‐Sosa, Gastélum‐Franco, Camacho‐Dávila, Torres‐Muñoz, & Nevárez‐Moorillón, 2010; Dunford & Vazquez, 2005). The main MOO constituents consist of carvacrol, thymol, β‐myrcene, α‐terpinene, ɤ‐terpinene, *p*‐cymene, and ceneol; components with antibacterial, antioxidant, antiviral, antifungal, and insecticidal properties (Ortega‐Nieblas et al., 2011; Silva‐Vazquez et al., 2017; Vazquez & Dunford, 2005).

The aim of this study was to evaluate the effect of a plant extract, oregano essential oil from Mexican oregano, *Lippia berlandieri* Schauer, as substitute for Mexican oregano powder on pork chorizo formulation and effects on preservation of physicochemical, textural, microbiological, antioxidant, and sensory characteristics during storage.

## 2 MATERIALS AND METHODS

### 2.1 Experimental design

A random complete design of two treatments was carried out in which Mexican oregano powder was replaced by essential oil from Mexican oregano *Lippia berlandieri* Schauer in the chorizo formulation: T1 = chorizo + 0.1% (w/w) MOP; T2 = chorizo + 0.1% (v/w) MOO. The MOP was purchased from Organización Soriana (Tiendas Soriana, S.A. de C.V., Nuevo Leon, Mexico). The MOO used in the study was purchased from Natural Solutions Company SMI (Jimenez, Chihuahua, Mexico). The essential oil chemical composition consisted of 60.0% carvacrol, 3.4% thymol, 16.1% cymene, 5.4% terpinene, 0.8% menthol, 0.3% cineole, 0.3% linalool, 0.3% limonene, and 0.09% myrcene. The *Lippia berlandieri* Schauer essential oil yields were approximately 4%, based on the feedstock weight. The MOO composition was analyzed by gas chromatography (PerkinElmer Clarus 600 and SQ8 GC/MS; PerkinElmer Inc., Waltham, MA, USA) according to the methods of Vazquez and Dunford (2005).

### 2.2 Chorizo preparation

Each treatment was formulated in two replicates of 2.0 kg each. The formulation was established according to González and Díez (2002), with some variation. The formulation (w/w) consisted of 75.0% lean pork meat, 18.0% pork back fat, 2.2% guajillo chili pepper powder, 0.1% paprika powder, 0.1% Mexican oregano powder, 0.5% garlic powder, 1.7% NaCl, and 2.4% acetic acid (white vinegar; Clemente Jacques, Sabormex S.A. de C.V., Mexico City, Mexico). The meat and pork back fat, maintained at 4.0°C, were ground through a 3/8‐in (9.5‐mm) grind plate using a TORREY^®^ grinder (Model MV‐22R‐SS; Grupo Torrey, S.A. de C.V., Nuevo Leon, Mexico). Next, powder ingredients were added and mixed manually for 8 min into the ground meat. For the treatment T2 preparation, MOO was added in place of the MOP. White vinegar was added and mixed manually for 5 min into the meat and until a homogeneous chorizo batter was obtained. The chorizo batter was placed in a TORREY^®^ mixer (Model MV‐25; Grupo Torrey, S.A. de C.V., Nuevo Leon, Mexico) for 25 min. Finally, the chorizo batter was encased in 3.0‐cm‐diameter cellulose synthetic casing and 15‐cm individual links were prepared from each treatment replicate. The chorizo links were stored at 4.0°C for evaluation at 1 and 7 d.

### 2.3 Physicochemical analysis

The pork chorizo pH was determined with a puncture electrode (Orion 3 Star Thermo Fisher Scientific, Pittsburgh, PA, USA). Chorizo color values were measured with a colorimeter (CR‐400 Konica Minolta^®^, Tokyo, Japan). Lightness (L*), redness (a*), yellowness (b*), Chroma (saturation index), and Hue angle were recorded. Total color change (∆*E*) and browning index (BI) were estimated according to Ledesma et al. (2016). The pH and color were measured in triplicate at four sections of the chorizo for each replicate per treatment (*n* = 24 per treatment). The colorimeter was standardized and calibrated using a white plate.

### 2.4 Shear force (SF) and texture profile analyses (TPA)

The SF and TPA of the chorizo were determined at 4°C with a TA.XT Plus texturometer (Stable Micro Systems, Surrey, UK) in eight sections per replicate of chorizo (two replicates per treatment; *n* = 16 per treatment). The SF was performed using a Warner–Bratzler shear blade with a triangular slot cutting edge. Ten cylindrical segments (3.0 cm diameter × 4.5 cm long) were used to evaluate the SF. The test conditions used in the instrument were a velocity of 2 mm/s pretest, 2 mm/s during the test, 10 mm/s post‐test, and a distance of 40 mm. The SF value was taken from the maximum point of the curve obtained from the test.

The TPA was determined in eight standardized cylinders per replicate (3.0 cm diameter × 2.5 cm high). A cylindrical piston (75 mm in diameter) was used to compress the sample during two test cycles, compressing the sample up to 60% from the original height within a time span of 5 s between cycles. Force–time curves of deformation were obtained from the conditions established in the texturometer. The velocities used were pretest 1.0 mm/s, during the test 5.0 mm/s, and post‐test 5.0 mm/s. The following parameters were obtained according to Bourne (1978): hardness (g), adhesiveness (g/s), springiness (mm), cohesiveness (dimensionless), gumminess (g), chewiness (g mm), and resilience (dimensionless).

### 2.5 Microbiological analysis

Microbiological counts were carried out according to Casquete et al. (2012) and NOM‐110‐SSA1 (1994) in triplicate on two sections at 1 and 7 d per replicate (*n* = 6 per treatment per day). A total of 10 g per sample were collected aseptically, transferred to sterile polyethylene bags to which was added 90 ml of sterile phosphate buffer (pH 6.5). Each sample was subjected to three 1.5‐min mixing cycles in a Stomacher (Seward Lab., London, UK). A 1‐ml portion was transferred onto a nutrient agar plate (gelatin peptone [5.0 g/L], meat extract [3.0 g/L], and agar [15.0 g/L]) (Becton Dickenson de México S.A. de C.V., Mexico State, Mexico). Inoculated plates were incubated at 30°C for 48 hr, and aerobic mesophilic bacteria colony counts were determined.

### 2.6 Antioxidant capacity

Antioxidants have been widely used for preservation of foods and feed (Silva‐Vazquez et al., 2017), and their effects are principally measured with antioxidant assays containing the radical 2,2‐diphenyl‐1‐picrylhydrazyl (DPPH) or 2,2′‐azino‐bis (3‐ethylbenzothiazoline‐6‐sulfonic acid) (ABTS), principally. The DPPH activity was determined according to method of Mielnik, Aaby, and Skrede (2003) with slight modifications. The chorizo samples were diluted 1:20 in ethanol (sample:ethanol). Fifty microliters of each diluted chorizo sample was added to 1 ml of DPPH in ethanol solution (25 mg/L). Reaction mixtures were incubated at 25°C for 20 min in darkness. Sample optical densities were measured in a spectrophotometer (Shimadzu UV‐VIS 1800, Kyoto, Japan) at 517 nm. All samples were measured at d 7 of storage in triplicate on two sections for each treatment (*n* = 6 per treatment).

### 2.7 Sensory evaluation

An affective sensory test of attributes was conducted at 1 and 7 d of storage to measure the level satisfaction of 30 consumers. Each consumer evaluated two chorizo slices (6 mm thickness) chosen at random. Samples for evaluation were maintained at 4°C and were presented on plastic Petri dishes. The attributes evaluated were red color, oregano odor, appearance, smoothness, and overall acceptability. A 7‐point hedonic scale was used in this test, where 7 = liked very much and 1 = disliked very much (Anzaldúa‐Morales, 1994; Meilgaard, Civille, & Carr, 2006).

### 2.8 Statistical analysis

An analysis of variance was performed using the proc GLM of SAS (2006) and the next model statistical: *y*_*ijk*_ **= μ + Ƭ_*i*_ + δ_*j*_ + (Ƭδ)_*ij*_ + Ԑ_*ijk*_, where *y*_*ijk*_ = physicochemical, textural, microbiological, antioxidant, and sensory variables evaluated over time; μ = general media; Ƭ_*i*_ = fixed effect of *i*‐th treatment (T1 and T2); δ_*j*_ = effect of *j*‐th evaluation day (1 and 7 d); (Ƭδ)_*ij*_ = effect of the interaction between the *i*‐th treatment and the *j*‐th day; Ԑ_*ijk*_ = random error normally independently distributed with media of zero and variance σ^2^ [Ԑ_*ijk*_ ~ NID (0, σ^2^)]. The statistical model of sensorial analysis considered a complete random block design, where each consumer was the block effect (β_*j*_) in each period. A significance level of *p* < .05 was used to assess significant differences between treatment means, days, and interaction. When the fixed effects and its interaction had significant effect, the means were compared using Adjust = Tukey (SAS 2006).

## 3 RESULTS

### 3.1 Physicochemical analysis

Table 1 shows the pH and color parameters of pork chorizo at 1 and 7 d. The pH, redness (a*), yellowness (b*), saturation index (Chroma), and browning index (BI) were affected statistically (*p* < .05) by the treatments and days of storage. At d 1, the pH was lower in T2 than in T1, however, was not different at d 7. Measurements of a* were different statistically (*p* < .05) at d 7 with a* for T2 being higher than in T1, as well as higher than T1 and T2 at d 1. Values for b*, Chroma, and BI were also higher at d 7 for T2 compared to T1. L*, Hue angle, and ∆*E* were not influenced statistically (*p* > .05) by treatments at each time and within treatments on different days. These results could indicate that 0.1% MOO can improve the chorizo color, because color variables presented high values in T2.

**Table 1:** Physicochemical parameters of pork chorizo sausage following treatment with Mexican oregano powder and *Lippia berlandieri* Schauer oregano essential oil and storage at 1 and 7 d

Days/Treatments	Variables							
	pH	L*	a*	b*	Hue	Chroma	BI	∆CT
Day 1								
T1	5.34^a,A^	35.83	23.11^a,B^	14.85^b^	33.12	27.53^a,A^	97.16^b^	63.90
T2	5.27^b,B^	36.66	23.13^a,B^	16.68^a^	35.79	28.60^a,B^	103.42^a^	63.48
Day 7								
T1	5.35^a,A^	36.83	22.67^b,B^	15.72^b^	34.58	27.71^b,A^	97.55^b^	62.98
T2	5.34^a,A^	37.10	25.27^a,A^	17.45^a^	34.74	30.79^a,A^	109.82^a^	64.09
SEM	0.02	0.41	0.50	0.56	1.07	0.52	2.97	0.04
RMSE	0.06	1.42	1.71	1.95	3.72	1.79	10.30	1.49
*p*‐Value								
Treatments (Ƭ_*i*_)	.0192	.1867	.0111	.0029	.1941	.0002	.0032	.4236
Days (δ_*j*_)	.0192	.0874	.0946	.1526	.8471	.0272	.2597	.7156
(Ƭδ)_*ij*_	.0581	.5059	.0126	.9356	.2478	.0579	.3186	.0838

T1: chorizo + 0.1% Mexican oregano powder; T2: chorizo + 0.1% *Lippia berlandieri* Schauer oregano essential oil; SEM, standard error of means; RMSE, root‐mean‐square error; Ƭ_*i*_: fixed effect of *i*‐th treatment (T1 and T2); δ_*j*_: effect of *j*‐th evaluation day (1 and 7 d); (Ƭδ)_*ij*_: effect of the interaction between the *i*‐th treatment and the *j*‐th day; L*: Lightness; a*: redness; b*: yellowness; Chroma: saturation index; ∆*E*, Total color change; BI, browning index.

Means (*n* = 24) within the same column and within each treatment and at different times with different superscripts (lower case) differ significantly when the *p*‐value of (Ƭ_*j*_) <.05.

Means (*n* = 24) within the same column, for all treatments and for all days, with different superscripts (upper case) differ significantly when the *p*‐value of (Ƭδ)_*ij*_ <.05.

### 3.2 Shear force (SF) and texture profile analyses (TPA)

Table 2 shows the SF and TPA of chorizo at 1 and 7 d of storage. Shear force was influenced statistically (*p* < .05) by time, with SF for T2 being higher at d 7 than at d 1. Hardness, springiness, gumminess, and chewiness were affected by interaction between treatments and days (*p* < .05), while adhesiveness and cohesiveness were influenced by storage days (*p* < .05) only. At d 1, hardness was different (*p* < .05) between chorizos treatment with T1 > T2; however, at d 7, the treatments were similar (*p* > .05), although T2 hardness was numerically higher than T1. Similar results by time occurred with springiness, gumminess, and chewiness when 0.1% OEO (T2) was used in the chorizo formulation.

**Table 2:** Shear force and texture profile analysis of pork chorizo sausage following treatment with Mexican oregano powder and *Lippia berlandieri* Schauer oregano essential oil and storage at 1 and 7 d

Days/Treatments	SF (g)	Texture profile analysis					
		Hard (g)	Adh (g s^−1^)	Sprin (mm)	Coh	Gum (g)	Chew (g mm)
Day 1							
T1	244.35^a,A^	1392.75^a,A^	−85.95^a,AB^	0.3675^a,B^	0.3545^a,AB^	491.45^a,A^	180.25^a,AB^
T2	216.99^a,B^	872.29^b,B^	−59.07^a,B^	0.3351^a,B^	0.3264^a,B^	284.38^b,B^	95.43^b,B^
Day 7							
T1	270.79^a,A^	1460.96^a,A^	−119.74^a,A^	0.4076^a,A^	0.3941^a,A^	550.46^a,A^	226.29^a,A^
T2	277.87^a,A^	1595.58^a,A^	−107.75^a,A^	0.4375^a,A^	0.3675^a,AB^	583.95^a,A^	254.35^a,A^
SEM	0.11	88.06	16.14	0.0009	0.02	26.74	15.09
RMSE	32.83	256.41	47.01	0.03	0.05	77.85	43.91
*p*‐Value							
Treatments (Ƭ_*i*_)	.3332	.0365	.2389	.6752	.1309	.0029	.0698
Days (δ_*j*_)	.0004	.0001	.0160	.0001	.0352	.0001	.0001
(Ƭδ)_*ij*_	.1314	.0008	.6474	.0054	.9902	.0001	.0008

T1: chorizo + 0.1% Mexican oregano powder; T2: chorizo + 0.1% *Lippia berlandieri* Schauer oregano essential oil; SEM, standard error of means; RMSE, root‐mean‐square error; Ƭ_*i*_, fixed effect of *i*‐th treatment (T1 and T2); δ_*j*_, effect of *j*‐th evaluation day (1 and 7 d); (Ƭδ)_*ij*_, effect of the interaction between the *i*‐th treatment and the *j*‐th day; SF, shear force; Hard, hardness; Adh, adhesiveness; Sprin, springiness; Coh, cohesiveness; Gum, gumminess; Chew, chewiness.

Means (*n* = 16) within the same column and within each treatment and at different times with different superscripts (lower case) differ significantly when the *p*‐value of (Ƭ_*j*_) <.05.

Means (*n* = 16) within the same column, for all treatments and for all days, with different superscripts (upper case) differ significantly when the *p*‐value of (Ƭδ)_*ij*_ <.05.

### 3.3 Microbiological analysis and antioxidant capacity

Microbial analysis and antioxidant capacity results of pork chorizo are presented in Table 3. Mesophyll counts were not different statistically between treatments (*p* > .05), but there was an effect (*p* < .05) with storage days, with values for T1 and T2, respectively, being higher at d 7 than at d 1. Treatments had no statistical effect (*p *> .05) on DPPH; however, pork chorizo treated with MOO (T2) had a slightly higher mean value compared to the control (T1).

**Table 3:** Microbiological analysis and antioxidant capacity of pork chorizo sausage following treatment with Mexican oregano powder and *Lippia berlandieri* Schauer oregano essential oil and storage at 1 and 7 d

Parameter/variable	Treatments		SEM	RMSE	*p*‐Value
	T1	T2			
Microbiologic analysis (log CFU/g)			0.05	0.11	<.0001
Day 1					
Mesophilic aerobes	4.19^B^	4.37^B^			
Day 7					
Mesophilic aerobes	4.73^A^	4.50^A^			
Antioxidant capacity (%)			0.76	1.70	.4082
DPPH	26.48	27.42			

CFU, colony‐forming units; DPPH, 1,1‐diphenyl‐2‐picrilhydrazil; T1: chorizo + 0.1% Mexican oregano powder; T2: chorizo + 0.1% *Lippia berlandieri* Schauer oregano essential oil; SEM, standard error of means; RMSE, root‐mean‐square error.

Means (*n* = 6) for mesophilic aerobes within the same column by treatment and with different superscripts (upper case) differ significantly (*p *< .05).

### 3.4 Sensory evaluation

The attributes of sensory evaluation were affected by treatments (*p* < .05) at 7 d only (Table 4), with T2 presenting a stronger oregano odor. Color, appearance, smoothness, and overall acceptability did not present differences (*p* > .05) between treatments and storage times.

**Table 4:** Sensory evaluation of pork chorizos at 1 and 7 d of storage using *Lippia berlandieri* Schauer oregano essential oil instead of Mexican oregano powder

Days/Treatments	Affective attributes				
	Redness	Oregano odor	Appearance	Smoothness	Overall acceptability
Day 1					
T1	5.97	5.43^a,AB^	6.07	5.63	6.03
T2	5.90	5.73^a,A^	5.80	5.83	5.90
Day 7					
T1	5.80	4.63^b,B^	5.73	5.83	5.73
T2	5.93	5.70^a,A^	5.97	5.73	6.27
SEM	0.17	0.22	0.19	0.18	0.18
RMSE	0.95	1.20	1.02	0.99	0.97
*p*‐Value					
Treatments (Ƭ_*i*_)	.8476	.0025	.9291	.7835	.2606
Days (δ_*j*_)	.7008	.0609	.6568	.7835	.8508
(Ƭδ)_*ij*_	.5645	.0841	.1845	.4107	.0625

T1: chorizo + 0.1% Mexican oregano powder; T2: chorizo + 0.1% *Lippia berlandieri* Schauer oregano essential oil; SEM, standard error of means; RMSE, root‐mean‐square error; Ƭ_*i*_, fixed effect of *i*‐th treatment (T1 and T2); δ_*j*_: effect of *j*‐th evaluation day (1 and 7 d); (Ƭδ)_*ij*_: effect of the interaction between the *i*‐th treatment and the *j*‐th day.

Means (*n* = 30) within the same column and within each treatment and at different times with different superscripts (lower case) differ significantly when the *p*‐value of (Ƭ_*j*_) <.05.

Means (*n* = 30) within the same column, for all treatments and for all days, with different superscripts (upper case) differ significantly when the *p*‐value of (Ƭδ)_*ij*_ <.05.

## 4 DISCUSSION

### 4.1 Physicochemical analysis

The pH result obtained at d 1 and 7 may be attributed to the MOO pH (4.49 ± 0.05), because oregano powder pH was higher (6.28 ± 0.02). Salgado et al. (2006) reported lower pH values in onion chorizo than those reported here, while Bozkurt and Bayram (2006) obtained similar values in respect to this study with 0.1% MOO from *Lippia berlandieri* Schauer. On the other hand, at d 7, Bozkurt and Bayram (2006) obtained similar values for L*, ∆*E*, and BI, but lower values for a* and b* of the spicy sausage sucuk during ripening. These authors indicated that the color is the most important quality attribute of sucuk, as it influences consumer acceptability. Therefore, the results presented for a* (significant), L*, Hue, and ∆CT (not significant) when MOO was used could indicate that MOO can be used to maintain the redness of pork chorizo, as well as to maintain lightness, saturation, and color change, potentially enhancing consumer preference. Finally, similar results for pH and color were obtained by Lorenzo, González‐Rodríguez, Sánchez, Amado, and Franco (2013) at d 20 of storage when examining the effects of 1 g/kg of grape seed or chestnut extract in cured chorizo sausage.

### 4.2 Shear force (SF) and texture profile analyses (TPA)

A possible accelerated change in the product structure over time in T1 could explain a low SF value at d 7. The TPA results suggest that *Lippia berlandieri* Schauer MOO can be used to conserve the chorizo texture during storage, without changes in product structure over time. Lorenzo et al. (2013) found at d 7 similar values in hardness, springiness, chewiness, and gumminess when testing 1 g/kg of grape seed or chestnut extract in cured chorizo sausage, but cohesiveness was similar at d 19 to that of the control treatment. Adhesiveness and cohesiveness values were low in T2 at d 1 and 7. These results could suggest a presentation of better quality with treatment of this chorizo with MOO. Similar results in hardness were obtained by Ledesma et al. (2016) comparing quality after storage for 7 d. Bozkurt and Bayram (2006) obtained similar results in hardness, gumminess, chewiness, and adhesiveness at 15 d with sucuk sausages, but springiness and cohesiveness were higher than those with chorizos in the current study following treatment with *Lippia* MOO.

### 4.3 Microbiological analysis and antioxidant capacity

With respect to microbial evaluation in pork chorizo in the current study, Porcella et al. (2001) found similar Enterobacteriaceae and *Lactobacillus* counts in the control treatment when evaluating soy protein isolate treatment in chorizos. In contrast, Casquete et al. (2012) obtained higher values for aerobic mesophilic bacteria on chorizos inoculated with autochthonous starter cultures with respect to the current study with 0.1% OEO. These authors suggested that differences in the counts were due to processing and the type of product. The MOO could allow increases in the mesophilic bacteria counts during storage or decreases in pathogen bacteria numbers. In the current study, the treatments were not different for mesophilic aerobes at 7 d, but T2 (MOO) gave low values. Hence, microbial counts did not increase after seven days in T2, which could be attributed to an effect of MOO. By comparison, T1 (control group) presented high values at 7 d and was higher than those at 1 d.

The search for alternative methods to retard oxidative processes in meat has led to research with alternative natural antioxidants (Broncano et al., 2012). In the current study, MOO was used in chorizo as a natural preserver followed by an evaluation its effect in the shelf life. It is possible that the differences found in antioxidant capacity were due to antioxidant activity of the total phenolic (e.g., carvacrol and thymol) content of MOO (García‐Iñiguez de Ciriano et al., 2009). Also, Broncano et al. (2012) found higher antioxidant capacity values when using isolated and identified antioxidant compounds with chorizos stored in the ripening chamber (11°C and 78% RH) for 1 month and 20 d. These contrasts could be due to the ripening process over 1 month and 20 d compared with the current study with MOO in pork chorizo stored for 7 d only. However, MOO application in the chorizo formulation could improve the antioxidant capacity; hence, the shelf life could be increased. In the current study, antioxidant capacity was not different at 7 d, but T2 presented high values.

### 4.4 Sensory evaluation

In contrast to the oregano odor obtained in pork chorizo, García‐Iñiguez de Ciriano et al. (2009) found no differences in odor, taste, and general appearance in dry fermented sausages with extracts of *Borago officinalis* (340 ppm) enriched with ω‐3 polyunsaturated fatty acids. However, these authors indicated that vegetable extracts can include compounds that can give special attributes to the final products. Lorenzo et al. (2013) found no differences for color, aroma, rancidness, taste, and overall acceptability in dry‐cured sausages with natural extracts. Furthermore, they showed that the addition of grape seed extract (1 g/kg) as a natural antioxidant improved the acceptability of dry‐cured sausages. This could explain the oregano odor acceptability in the treatment with Mexican oregano oil that improved this sensory property. Furthermore, the stability found with color, appearance, smoothness, and acceptability could be considered stable because T2 (MOO) was not different from T1 (control) at 7 d of storage.

## 5 CONCLUSIONS

Essential oil (0.1%) of Mexican oregano, *Lippia berlandieri* Schauer, in chorizo formulation improved color parameters, especially red color, browning index, textural profile analysis, and sensory characteristics. Additionally, the mesophilic microbial counts were similar for the two treatments following storage for 7 d. Results of this study demonstrate the potential usefulness of *Lippia berlandieri* Schauer MOO to enhance quality, sensory characteristics, and shelf life of pork chorizo sausage.

## CONFLICT OF INTEREST

The authors declare that there is no conflict of interests.

## ETHICAL REVIEW

This study was approved by The Postgraduate and Research Subdirectorate‐Facultad de Agronomia, Universidad Autonoma de Nuevo Leon (number 051/16).
